# Pathophysiology and treatment of patients with beta-thalassemia – an update

**DOI:** 10.12688/f1000research.12688.1

**Published:** 2017-12-20

**Authors:** Eitan Fibach, Eliezer A. Rachmilewitz

**Affiliations:** 1 Department of Hematology, Hadassah – Hebrew University Medical Center, Jerusalem, Israel; 2 Department of Hematology, The Edith Wolfson Medical Center, Holon, Israel

**Keywords:** Thalessemia, hemolytic anemia, β-globin

## Abstract

Thalassemia (thal) is an autosomal recessive, hereditary, chronic hemolytic anemia due to a partial or complete deficiency in the synthesis of α-globin chains (α-thal) or β-globin chains (β-thal) that compose the major adult hemoglobin (α _2_β _2)._ It is caused by one or more mutations in the corresponding genes. The unpaired globin chains are unstable; they precipitate intracellularly, resulting in hemolysis, premature destruction of red blood cell [RBC] precursors in the bone marrow, and a short life-span of mature RBCs in the circulation. The state of anemia is treated by frequent RBC transfusions. This therapy results in the accumulation of iron (iron overload), a condition that is exacerbated by the breakdown products of hemoglobin (heme and iron) and the increased iron uptake for the chronic accelerated, but ineffective, RBC production. Iron catalyzes the generation of reactive oxygen species, which in excess are toxic, causing damage to vital organs such as the heart and liver and the endocrine system.

Herein, we review recent findings regarding the pathophysiology underlying the major symptoms of β-thal and potential therapeutic modalities for the amelioration of its complications, as well as new modalities that may provide a cure for the disease.

## Introduction

Thalassemia (thal) is an autosomal recessive hereditary chronic hemolytic anemia due to a partial or complete deficiency in the synthesis of α-globin chains (α-thal) or β-globin chains (β-thal) which compose the major adult hemoglobin (HbA), a tetramer of α _2_β _2_. It is caused by one or more of several hundred mutations in the corresponding genes. The unpaired globin chains are unstable; they precipitate intracellularly, resulting in hemolysis, premature destruction (by apoptosis) of red blood cell (RBC) precursors in the bone marrow, and a short life-span of mature RBCs in the circulation. The breakdown products of Hb, heme and iron, catalyze chemical reactions that generate free radicals, including reactive oxygen species (ROS), which in excess are toxic, causing damage to vital organs such as the heart and liver and the endocrine system ^1^.

Thalassemia is the most common monogenic inherited disease worldwide. Historically, it originated and spread around the Mediterranean, the Middle East, and Southeast Asia, coincidental with the occurrence of malaria (carriers of the thal genes are considered to be resistant to the malaria parasite) ^2^. Today, because of vast migration, thal patients are present around the globe ^3^ and their incidence increases steadily.

Beta-thal is classified into three main subgroups based on their clinical expression: major, intermedia, and minor. β-thal major presents itself within the first 2 years of life with severe anemia, poor growth, and skeletal abnormalities and requires regular, lifelong blood transfusions. β-thal intermedia requires only periodic blood transfusions, while β-thal minor does not require a specific treatment. Alpha-thal presents with moderate anemia when there is a significant lack of synthesis of α-globin chains (HbH disease).

There are several combinations of β-thal with other diseases associated with abnormal β-globin, such as HbE and HbS (sickle cell disease) that can be expressed clinically with severe anemia. A combination of β-thal with α-thal results in a milder disease, most likely owing to the less severe α:β imbalance ^4^.

Thanks to the significant improvement in therapy, patients with β-thal may reach an advanced age. This is associated with clinical symptoms that are the consequence of the disease itself and the treatment modalities.

Herein, we review recent findings regarding the pathophysiology underlying the major symptoms of β-thal and potential therapeutic modalities for the amelioration of its complications, as well as new modalities that may provide a cure for the disease.

## Chronic anemia – RBC transfusions

The basic clinical symptom of β-thal is chronic anemia—reduced number of RBCs and their Hb content, resulting from deficiency in Hb production and hemolysis. Chronic anemia is treated by RBC transfusion—in severe cases, every 2 weeks—which affects the patient’s quality of life, may cause recurrent infections and immune reactions, and—above all—iron overload (IO). Iron deposition in vital organs, through the generation of ROS, is a major cause of morbidity and mortality, especially among elderly patients ^5^.

Potential developments in the field of transfusion (beyond the scope of this review) include, among others, conditions of storage ^6^, *ex vivo* production of stem cell-derived RBCs ^7^, and transfusion of cell-free Hb ^8^.

## Iron overload

The iron status reflects the balance among dietary iron uptake, its storage and mobilization, and its utilization. Iron overload is a common and serious problem in thal ^9^, as well as in other hereditary and acquired hemolytic anemias ^10^. The main causes of IO in thal are Hb instability, RBC transfusions, and increased iron absorption from the gastrointestinal tract.

Normally, 1–2 mg of iron is absorbed from the diet per day, with an equivalent amount lost by the turnover of gastrointestinal tract epithelial cells. The body has no mechanism for disposing of excess iron ^11^; therefore, in thal and other transfusion-dependent anemias, IO may accumulate in a relatively short time (transfusional IO). An RBC transfusion requirement of two units (200–250 mg of iron per unit) per month will result in over 20 g of excess body iron in 4 years ^12^.

Most of the iron in the body is bound to other molecules. In the plasma, it is bound to transferrin ^13^. When the transferrin iron-binding capacity is saturated (>80%), non-transferrin-bound iron (NTBI) forms appear ^14^. Most of the iron gets into cells through their surface transferrin receptor (TfR1) ^15^, but a small fraction is taken up by non-transferrin pathways ^16^. In erythroid cells, the incoming iron is mainly incorporated into heme to form the Hb molecule or is stored in ferritin ^17^. A small fraction remains free or loosely bound to other compounds as the labile iron pool (LIP) ^18^. The LIP has been suggested as a low-molecular-weight intermediate or transitory pool between extracellular iron and intracellular firmly bound iron ^19^. The intracellular LIP is redox active, catalyzing the Fenton and Haber-Weiss reactions that generate ROS ^20^. Excess ROS are cytotoxic through their interaction with cellular components, such as DNA, proteins, and lipids, causing damage to vital organs ^21^.

### Removal of excess iron

Repeated bleeding (phlebotomy) is used to remove excess iron in patients with normal Hb levels, such as in patients with hereditary hemochromatosis, where IO is caused by mutations in the iron homeostasis system ^22^. Patients after hematopoietic stem cell (HSC) transplantation (HSCT) who had IO prior to transplantation due to multiple transfusions may also benefit from this treatment ^23^.

Most other IO patients are anemic (Hb <10 g/dL) and, therefore, particularly those who are transfusion dependent, will require iron chelation therapy in order to normalize their iron level (a transferrin saturation of <50% and serum ferritin <500 ng/mL). Iron chelators remove excess iron from the plasma and the cells by binding the labile, chelatable iron, thus facilitating its excretion through the urine and feces.

Three iron chelators are currently in clinical use. Deferoxamine, the first to be used clinically, is given by a slow, continuous, subcutaneous, overnight infusion through a portable pump. While its side effects are minimal, its mode of administration results in low compliance ^24^. Deferasirox, the first effective oral chelator, is given at 20–30 mg/kg once/day. Adverse effects (occurring in approximately 10% of patients) include nausea, abdominal pain, diarrhea, and rash as well as liver and kidney dysfunction. A new formulation of film-coated tablets provides better patient compliance, since it can be swallowed with a light meal without the need to disperse the tablet into a suspension prior to consumption ^25^. Deferiprone is an oral iron chelator effective in removing excess iron from the organs and mainly from the heart. The main potential complication is neutropenia that may rarely be followed by agranulocytosis. A liquid formulation has been recently introduced ^26^.

The efficacy of chelation may be improved by the use of a combination of chelators. Thus, deferiprone may mobilize iron from tissues into the circulation, where deferoxamine binds and facilitates its excretion in the urine (the “shuttle mechanism”) ^27^.

An additional potential approach to reduce iron load, especially NTBI, is the administration of exogenous iron-free (apo)transferrin through the down regulation of TfR1 ^28^.

In addition to free iron, some iron-containing compounds that are elevated in the plasma of thal patients due to hemolysis, such as free hemin and Hb, are of considerable toxicity. These compounds are neutralized by their scavengers, hemopexin and haptoglobin, respectively. In thal, these proteins are reduced, leaving free, un-neutralized hemin and Hb. The administration of hemopexin/haptoglobin may be suggested to reduce iron toxicity ^29^.

### Modulation of iron absorption

Iron homeostasis is tightly regulated, mainly by hepcidin, a 25-amino-acid peptide which inhibits iron absorption and distribution. It binds to ferroportin, a surface iron exporter on absorptive enterocytes, macrophages, hepatocytes, and placenta cells. This binding induces ferroportin to be internalized and degraded, decreasing, consequently, the export of iron from these cells ^30^.

The level of hepcidin depends mainly on its rate of production in the liver, which is modulated mainly by the iron status and requirement. Iron loading increases hepcidin production, resulting in reduced intestinal iron absorption while iron deficiency has an opposite effect ^30^.

In β-thal, in spite of IO, hepcidin production is generally low, and consequently iron absorption is high ^31^. The reason for this anomaly is the inhibition of hepcidin gene expression caused by two factors. (A) High iron demand by the chronic stress erythropoiesis (see below) ^32^. Soluble factors such as the growth differentiation factor (GDF)-15 ^33^, twisted gastrulation protein homolog 1 ^34^, soluble transferrin receptor ^35^, and erythroferrone ^36^, which are overproduced by the proliferating erythroid precursors, inhibit hepcidin expression. (B) Oxidative stress (see below) due to the inactivation of transcription factors, including CCAAT/enhancer-binding protein a (C/EBPa) and signal transducer and activator of transcription 3 (STAT3) ^37^, histone deacetylase activation ^38^, and hypoxia-inducible factors ^39^. The decrease in hepcidin production is, however, balanced by other conditions prevalent in β-thal, including blood transfusions and inflammation that increase hepcidin production. In the latter case, pro-inflammatory cytokines, such as interleukins 6 and 1, turn on the hepcidin gene promoter by activating the STAT3 pathway ^40^.

Administration of hepcidin or stimulating its expression could improve IO by decreasing iron absorption. This was demonstrated in murine models of β-thal intermedia by (A) minihepcidins, small peptides that mimic hepcidin activity and act as agonists ^41^, (B) inhibition of negative regulators of matriptase-2 (TMPRSS6), a key regulator of hepcidin production, with silencing RNAs or antisense oligonucleotides ^42^, (C) exogenous transferrin through downregulation of TfR1 ^28^, and (D) inhibition of erythroferrone ^43^.

## Dyserythropoiesis

The chronic anemia and its associated hypoxia in thal stimulate increased production of RBCs (chronic stress erythropoiesis). This is mediated by overproduction of erythropoietin (EPO), the main erythropoietic stimulating hormone, and other factors, such as members of the transforming growth factor (TGF)-β and activin receptor-II (ActR-II) trap ligands ^44^. Binding of EPO to its surface receptor on erythroid precursors activates transduction pathways, including Jak2/Stat5, which inhibit apoptosis and induce proliferation and differentiation of the developing cells. However, this attempt is futile (“ineffective erythropoiesis”) due to oxidative stress-increased apoptosis and abortive differentiation.

Several therapeutic modalities aimed at reducing the dysery-thropoiesis in thal are currently under study and described below.

### Activin receptor-II trap ligands

Luspatercept and Sotatercept, compounds that bind to trap ligands and GDF-11, developed in animal models, are currently in clinical trials ^44^. They prevent activins binding to ActR-II and the activation of the Smad 4-dependent signaling pathway, improving erythroid maturation and RBC production. A phase 3, multicenter, multinational study with luspatercept is ongoing in β-thal and HbE/β-thal subjects, with encouraging preliminary results ^45^.

### JAK2 inhibitors

The EPO signaling of erythropoiesis involves Jak2 phosphorylation. Beta-thal mice have elevated EPO levels associated with increased Jak2 phosphorylation, resulting in ineffective erythropoiesis and extramedullary hematopoiesis ^46^. Jak2 inhibitors effectively reduce splenomegaly in such mice. Several Jak2 inhibitors have been developed with beneficial results in patients with myelofibrosis and Jak2-related polycythemia vera ^47^. Jak2 inhibitors could be also beneficial for non-transfusion-dependent thal patients with splenomegaly ^48^.

### Induction of the Hsp70 chaperone machinery

The heat shock protein 70 (Hsp70) is a molecular chaperone needed for normal termination of erythropoiesis ^49^. It is predominant in the late erythroid precursors when it is translocated to the nucleus and protects the globin transcription factor-1 (GATA-1), the principal transcriptional factor for erythropoiesis, from caspase-3 cleavage ^50^. In β-thal major, HSP70 is sequestrated in the cytoplasm, leaving GATA-1 unprotected from cleavage, resulting in end-stage maturation arrest and apoptosis ^51^. Exportins, such as XPO1, are factors that control the nucleocytoplasmic trafficking of proteins and RNAs. The XPO1 inhibitors leptomycin B and KPT 251, recently tested in erythroid progenitors from β-thal major patients, demonstrated induction of HSP70 nuclear localization, GATA-1 expression, and improved terminal erythroid differentiation ^52^.

### Reducing α-globin synthesis

The key pathophysiological mechanism leading to the ineffective erythropoiesis in β-thal is the continuous production and accumulation of free excess α-globin in the erythroid precursors. Indeed, reduction in α-globin chain output through co-inheritance of α-thal ameliorates the disease phenotype in patients with β-thal ^53^. The challenge here is selective silencing of the α-globin expression to an appropriate degree to be useful for patients with β-thal ^54^. Plausible approaches include post-transcriptional silencing through RNA interference (RNAi) using small interfering RNAs, short hairpin RNA, epigenetic drug targeting to alter the chromatin environment of the α-globin genes, and genome editing to disrupt the expression of the α-globin genes ^55^.

## Oxidative stress

Although oxidative stress is not the primary etiology of thal, it plays a major role in its pathophysiology. The oxidative status of cells is regulated by the equilibrium between oxidants, such as the reactive oxygen species (ROS) that are produced mainly as byproducts of cellular respiration, and anti-oxidants, such as reduced glutathione. A balanced oxidative state is crucial for normal physiology. ROS serve as regulators in many processes, including proliferation and differentiation of the erythroid precursors. When this balance fails, such as in many pathological processes, oxidative stress ensues. The excess ROS bind to cell components such as the DNA, proteins, and membrane lipids, leading to cytotoxicity ^21^. In β-thal, oxidative stress is mainly the consequence of the unstable Hbs (hemichromes) and IO and it mediates many of its symptoms due to oxidative damage to RBCs (anemia), platelets, (hypercoagulable state) and leukocytes (recurrent infections) as well as cells in various vital organs (heart and liver) and the endocrine glands ^21^.

Oxidative stress may be ameliorated by endogenous and exogenous antioxidants. Their effects include scavenging and inactivating ROS and correcting their damage to cellular components. Many antioxidants are supplied by nutrition. A "Mediterranean diet" and moderate wine consumption are thought to have a protective effect ("the French Paradox") due to their high content of antioxidants ^56, 57^. Antioxidants can also be taken as food additives, either as pure compounds, such as vitamins C and E and Q10, or as crude extracts, such as the fermented papaya preparation and curcumin ^9^. Using such additives succeeded in ameliorating various parameters of oxidative stress in thal, but a clear clinical benefit, such as reducing transfusion dependence, was less successful. Effective outcome may require a combination of drugs, especially those affecting both the oxidative stress and the IO.

A newly discovered therapeutic target is the interaction between the oxidative state and the processes of erythroid cell proliferation and differentiation, which, as mentioned above, is defective (dyserythropoiesis) in β-thal. Several agents have been tested recently, especially in murine models of β-thal intermedia (β-thal mice). Although promising, the findings of these studies need careful interpretation, given the difference in globin gene composition and erythroid differentiation between human and mouse ^58^.

The transcription factor forkhead box O3 (Foxo3) is a key player in the development of erythroid precursors. At early stages, it is phosphorylated by proteins of the EPOR-PI3K/AKT/mTOR pathway, translocated out of the nucleus, and remains inactivated. At late stages, it is relocated into the nucleus, gets activated, and induces the production of antioxidants that neutralize ROS to allow efficient erythropoiesis ^59, 60^.

In β-thal mice, Foxo3 is downregulated in late erythroid precursors owing to hyper-activation of the EPOR-PI3K/AKT/mTOR pathway, which leads to oxidative damage and ineffective erythropoiesis ^61^.

Rapamycin, an mTOR inhibitor, has been shown in β-thal mice to improve erythroid cell maturation, β-globin production, and anemia through Foxo3 activation ^61^. In another study, rapamycin increased γ-globin expression and HbF production in cultured erythroid precursors from β-thal intermedia patients ^62^. Another Foxo3-activating agent is resveratrol (3,5,4’-trihydroxy-trans-stilbene), a non-flavonoid polyphenol that upregulates antioxidants. The use of resveratrol in β-thal mice has been shown to increase RBC survival and Hb levels and reduce reticulocyte count ^63^. In contrast, a study in double mutant Foxo3 ^–/–^/Th3/1 mice showed that a loss of Foxo3 leads to improved early erythropoiesis ^64^.

Consequently, further laboratory and clinical investigations are required in this field.

The eukaryotic initiation factor 2 (eIF2) is a factor required for the initiation of translation through the binding of tRNA to the ribosomes. In the erythroid precursors, its activity is regulated by a mechanism involving phosphorylation at its α-subunit by heme-regulated eIF2a kinase (HRI). Stress (such as heme deficiency and oxidative stress) ^65^ during the late stage of erythroid differentiation activates HRI that coordinates the syntheses of heme and globin. Furthermore, the phosphorylated α-subunit of eIF2 has been demonstrated to turn on the activating transcription factor 4 (ATF4) to diminish oxidative stress in erythroid precursors ^66, 67^.

This kinase has been found to be decreased in β-thal mice, leading to embryonic lethality ^68^. Salubrinal, a selective inhibitor of eIF2aP dephosphorylation, has been found to augment the HRI signaling pathway and to reduce the production of hemichromes in β-thal erythroid precursors ^67^. It has also been shown to increase HbF production with a concomitant decrease of HbA in differentiating human CD34 cells by a post-transcriptional mechanism ^69^. These findings provide the basis for manipulating the HRI-eIF2aP signaling pathway for the treatment of β-thal.

Peroxiredoxin-2 (Prx2) is an essential antioxidant protein that scavenges and inactivates ROS throughout erythropoiesis. It has been found to be upregulated during both murine and human β-thal ^70^. Knockout of Prx2 in β-thal mice worsened their phenotype, while administration of fused recombinant PEP1Prx2 ameliorated their symptoms, with activation of the Erk signaling pathway towards Tfr2 and the Sma and Mad (SMAD) system ^71^.

Heme oxygenase-1 (HO-1) is an enzyme that catalyzes the degradation of heme in response to stress, such as oxidative stress or hypoxia, both of which occur in β-thal ^72^. Its expression has been found to be augmented in EPO-dependent fetal liver erythropoietic cells from β-thal mice. Administration of tin protoporphyrin IX, an HO-1 inhibitor, improved ineffective erythropoiesis and Hb levels, and decreased spleen size and liver iron ^73^.

## Stimulation of HbF production

During prenatal life in humans, the major Hb is fetal Hb (HbF), a tetramer of α- and γ-globin (α _2_γ _2_) which is replaced during the first year of life by HbA (α _2_β _2_) (Hb switching). Thus, the clinical features of β-hemoglobinopathies, including β-thal, are not apparent at birth; only as HbF levels wane are the symptoms manifested ^74^. Patients with β-thal produce high but variable levels of HbF compared to normal individuals. High levels of HbF ameliorate the severity of the disease, mainly by reducing the surplus of α-globin chains.

These findings have motivated the research of the mechanisms of Hb switching as well as for pharmacological and gene modification modalities to reactivate the expression of the γ-globin genes and production of HbF ^75^.

### Pharmacological approach

Various compounds have been tested *in vitro* and in animal models for their capacity to reactivate the γ-globin genes ^76^. Currently, the only compound in clinical use is hydroxyurea, an S-phase cell cycle inhibitor. However, its mechanism of action on HbF remains elusive, a subset of patients are resistant, its effect in β-thal is inferior compared to that of sickle cell disease, and being myelosuppressive necessitates careful monitoring of patients ^77^.

New agents include those that affect chromatin regulators (such as decitabine on DNA methylation and histone deacetylase inhibitors) and others that affect DNA-binding transcription factors.

### Gene modification approach

The patient's HSCs are subjected to gene editing *ex vivo* and then returned to the patient for reconstitution ^78, 79^.

Increased production of γ-globin has been accomplished using lentiviral vectors that express a zinc finger protein which interacts with the promoter of the γ-globin gene ^80^ or by carrying microRNAs that silence its repressors ^81, 82^.

Two potent transcriptional repressors of γ-globin, BCL11A and ZBTB7A, have been identified. They act with additional *trans*-acting epigenetic repressive complexes, lineage-defining factors, and developmental programs to silence the γ-globin genes by working on *cis*-acting sequences at the globin gene loci. Inhibition of these repressors could reactivate γ-globin production in adult patients.

Most of the studies targeted BCL11A. Its inhibiting antisense oligonucleotides were administered in an erythroleukemia series expressing BCL11A and Krüppel-like factor 1 (KLF1) ^83, 84^. KLF1 activates the expression of the β-globin gene and it plays a role in the transcriptional silencing of the γ-globin gene, possibly through BCL11A ^85, 86^.

Genome editing of the promoter of BCL11A can be accomplished by several nucleases, such as engineered zinc finger nucleases (ZFNs), transcription activator-like effector nucleases (TALENs), and clustered regularly interspaced short palindromic repeats linked to Cas9 nucleases (CRISPR-Cas9) ^87^. It has been shown recently that ZFN-driven BCL11A enhancer ablation leads to increased production of HbF in erythroid precursors derived from β-thal HSCs, which could be used for autologous transplantation ^88^. A similar effect has been achieved with CRISPR-Cas9-mediated BCL11A enhancer inactivation in a human adult-stage erythroid cell line ^89^.

BCL11A has important roles in physiology; therefore, reducing its expression *in vivo* requires novel vectors that can restrict its effect to the erythroid lineage ^82^. Dissection of the erythroid-specific enhancer down to a small region in the gene offers such possibility ^90^. The same applies to other factors, like KLF1 and MYB, which are involved in HbF production.

One could also consider de-repressing γ-globin expression by forcing interaction of the β-locus control region with the γ-gene using a synthetic DNA-binding protein ^91, 92^.

## Gene therapy

Gene therapy involves *in vivo* genetic manipulation of the autologous HSCs, which are then transplanted to the patient for reconstitution ^78, 79^. This approach has focused on two areas. (A) Increasing the production of γ-globin by the addition of its gene, overexpression of its endogenous activating transcription factors, and silencing of its repressors, as discussed above. (B) Increasing the production of β-globin by the addition of a normal gene or correction of the mutated gene. Studies of gene therapy have utilized mainly lentivirus vectors in experimental systems, including cultured CD34 HSCs from β-thal patients and β-thal mouse models. Yet the safety profile of such technologies is still uncertain.

From the current gene-modifying approaches, only β-globin addition has been tried in β-thal patients. Transfusion-dependent βE/β ^0^ patients have been transplanted with autologous CD34 progenitor cells transduced *ex vivo* with lentiviral β-globin vectors. To date, there are a total of seven patients who have been treated with encouraging results in terms of engraftment and transfusion independence; long-term follow up will clarify the possible insertional mutagenesis issues ^93, 94^.

Phase 1 clinical trials have been initiated in order to assess these issues. Early phase, open label clinical trials of LentiGlobin BB305 will assess its efficacy and safety in patients with β-thal major or sickle cell disease. Safety and tolerability of autologous CD34 HSCs transduced with TNS9.3.55 or GLOBE lentiviral vectors are also being assessed in ongoing trials. Preliminary data from the latter trial have been recently presented regarding three patients with β-thal major. All patients showed a satisfactory engraftment, with mild and reversible adverse events ^95^.

Genomic editing has been demonstrated to modify the β-globin gene. Thus, TALEN-mediated gene correction has been used in induced HSCs from β-thal patients ^96^.

## Allogeneic hematopoietic stem cell transplantation

Allogeneic HSCT (allo-HSCT) is currently the only definitive cure for transfusion-dependent young patients before the development of IO-related tissue damage ^97^. β-thal major patients with good risk features have a >90% chance of a successful outcome ^98, 99^, but allo-HSCT in high-risk patients is challenging because of graft rejection and transplant-related mortality ^14^. Novel modified or reduced-intensity conditioning regimens are being evaluated in an attempt to improve the outcome in such patients with favorable results ^100– 102^.

Traditionally, fully matched human leukocyte antigen (HLA)-identical siblings have been used as donors, but matched unrelated donors have also been tried in low-risk patients. Bone marrow is the preferable source of HSCs, but HSCs from the peripheral blood and cord blood are also being tried in low-risk cases.

## Prevention

In spite of the advent of therapeutic modalities that alleviate the symptoms and may even cure the disease, the incidence of affected newborns is expected to increase. Most of the new cases are in underdeveloped countries where the standard of medical care is low and in communities where consanguinity is high. Therefore, the prevention of the homozygous state presents an important endeavor. Comprehensively preventive programs involve carrier detections, molecular diagnostics, genetic counseling, and prenatal diagnosis. Currently, prenatal diagnosis is performed, for couples at risk, by obtaining fetal material by chorionic villus sampling in the first trimester and by amniocentesis or cordocentesis in the second trimester. An additional procedure for obtaining fetal material is aspiration of celomic fluid (celocentesis) followed by selection of embryo-fetal erythroid precursors by an anti-CD71 MicroBeads method or by direct micromanipulator pickup of the cells selected on the basis of their morphology ^103^. Molecular analysis, aimed at the detection of mutations in the globin genes, is then performed ^104^.

Recently, the possibility of safer and cheaper prenatal diagnosis procedures emerged. Fetal-derived genetic material (cells or cell-free DNA) is obtained from the maternal blood and tested. These non-invasive procedures that present no risk to the fetus and reduce cost (no special procedures and staff are required for sampling) may allow future screening for thal as well as other genetic diseases ^105^.

Indeed, non-invasive prenatal testing using maternal plasma cell-free DNA has already been applied for screening for common chromosomal aneuploidies. Progress has also been made in screening for monogenic diseases, using thal as a model disease. One approach focuses on the detection or exclusion of paternally inherited fetal mutations that are absent from the mother's genome. Testing maternally inherited mutations requires highly sensitive DNA quantification. The relative mutation/haplotype dosage approach might detect fetal mutations even when the parents share the same mutation.

Another approach is pre-implantation genetic diagnosis of cells (usually single cells) that had been biopsied from oocytes/zygotes or embryos generated by *in vitro* fertilization. With respect to thal, this technique aims at giving birth to an unaffected newborn, and, when relevant, for cord blood cells compatible with an existing affected sibling to support HSCT. Pre-implantation diagnosis precludes the need for abortion ^106^.

## Conclusion

**Figure 1.**
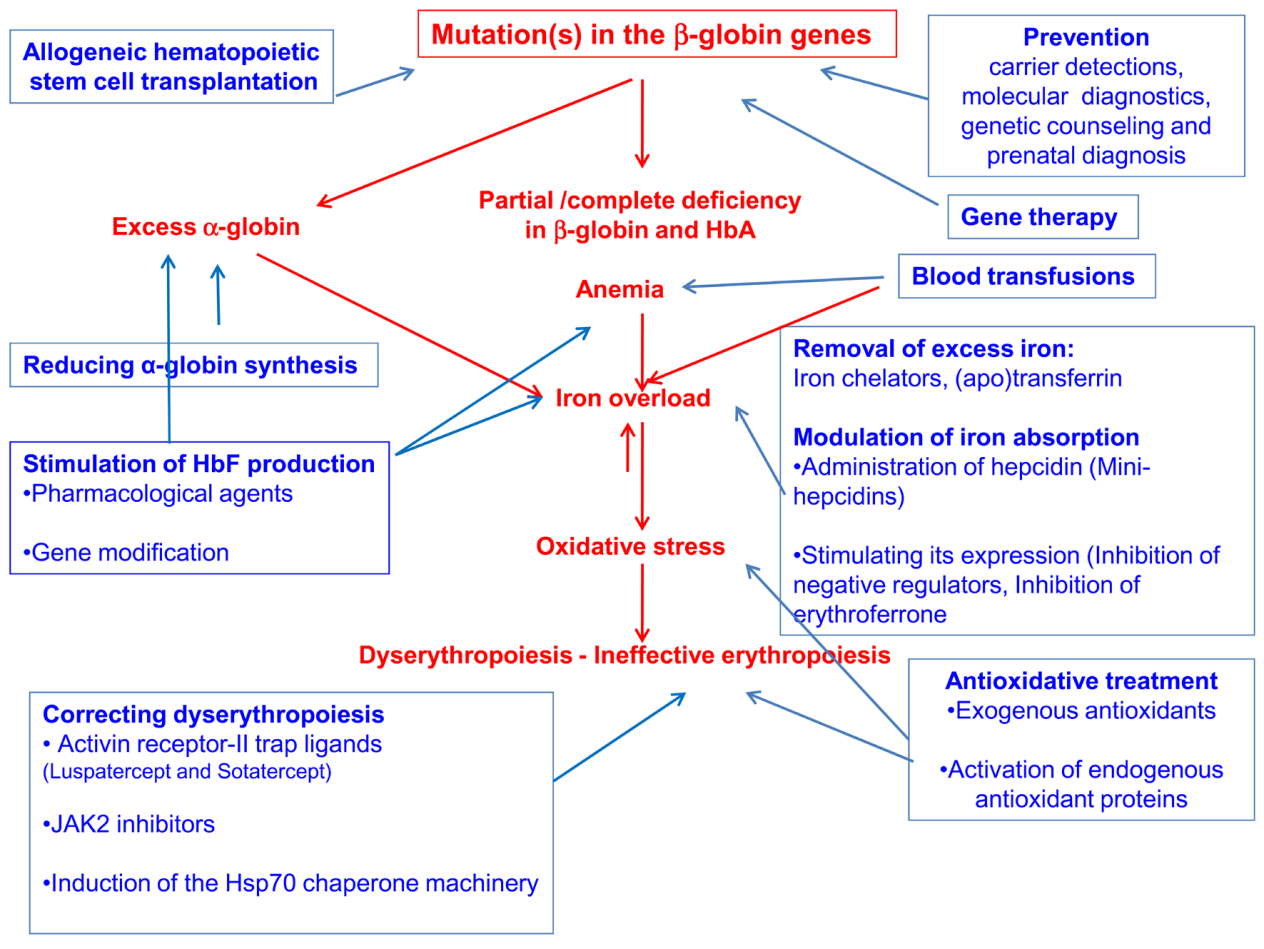
Beta-thalassemia: causes, symptoms, and therapeutic modalities. Causes and symptoms are marked in red; therapeutic modalities are marked in blue.

Beta-thalassemia is caused by mutations in the β-globin gene, resulting in partial or complete deficiency of its product. This deficiency and the accompanying excess of the unmatched α-globin chains result in oxidative stress, dyserythropoiesis, and chronic anemia. The main therapeutic modality is blood transfusion that improves the anemia but exacerbates IO. To date, the only curative measure is allo-HSCT. New modalities, aimed at various targets, are being developed. These include the means to stimulate the synthesis of γ-globin and reduce the synthesis of α-globin, as well as the iron excess, oxidative stress, and dyserythropoiesis. Attempts to increase β-globin synthesis focus on gene manipulation. However, the most likely approach to reduce the patients' load is efficient prevention: carrier detection, prenatal diagnosis, and genetic counseling ( Figure 1).
